# A Design and Application of Municipal Service Platform Based on Cloud-Edge Collaboration for Smart Cities

**DOI:** 10.3390/s22228784

**Published:** 2022-11-14

**Authors:** Jingmin Yang, Trong-Yen Lee, Wen-Ta Lee, Li Xu

**Affiliations:** 1 Department of Electronic Engineering, National Taipei University of Technology, Taipei 10667, Taiwan; 2 Fujian Provincial Key Laboratory of Network Security and Cryptology, Fujian Normal University, Fuzhou 350007, China

**Keywords:** cloud computing, cloud-edge collaboration, edge computing, evolutionary equilibrium, Nash equilibrium, smart cities

## Abstract

Information and Communication Technology (ICT) makes cities “smart”, capable of providing advanced municipal services to citizens more efficiently. In the literature, many applications of municipal service platform based on cloud computing and edge computing have been proposed, but the reference model and application instance based on cloud-edge collaboration specially for municipal service platform is rarely studied. In this context, this paper first develops a reference model, including resource collaboration, application collaboration, service collaboration, and security collaboration, and discusses the main contents and challenges of each part. Then, aiming at the problem of computing and communication resources allocation in the cloud-edge collaboration, a game-theory-based dynamic resource allocation model is introduced. Finally, an e-government self-service system based on the cloud-edge collaboration is designed and implemented. The cloud side is a cloud computing server, and the edge side are the self-service terminals integrating various edge computing devices with Artificial Intelligence (AI) embedded. The experimental results show that the designed system combines the advantages of cloud computing and edge computing, and provides a better user experience with lower processing latency, larger bandwidth, and more concurrent tasks. Meanwhile, the findings show that the evolutionary equilibrium and the Nash equilibrium are the optimal solutions, respectively.

## 1. Introduction

According to the 2019 revision of world population prospects released by United Nations Department of Economic and Social Affairs (UNDESA) in 2020, The world’s population is expected to grow from 7.7 billion in 2019 to 8.5 billion in 2030 (10% increase), and further to 9.7 billion in 2050 (26% increase). Furthermore, the urban areas are expected to absorb almost all future growth in the world population [1]. Although the rapid urban growth presents an important opportunity to enjoy the demographic dividends, it also brings many challenges to the implementation of urban development, such as safety, sustainability and resilience [2]. For example, Corona Virus Disease 2019 (COVID-19), which broke out suddenly at the end of 2019, quickly spread to major cities around the world. The Chinese city of Wuhan adopts advanced ICT to make it more smart, which has effectively helped in containing the spread of the COVID-19 [3]. In 2021, a research work points out that “smart cities” is a global adopted solution to the urbanization challenges [4]. It is worth affirming that smart cities create a more secure, sustainable, prosperous and resilient future for citizens through advanced ICT infrastructure.

**Figure 1:**
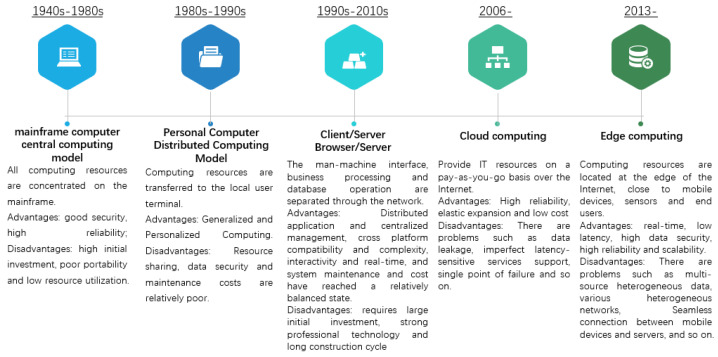
The development road map of the ICT’s main computing models.

The computing model of ICT develops with the prosperity of smart cities. To be exact, the original driving force is the rapid growth of urban population, which brings new application scenarios and needs, such as citizens’ safety, convenient transportation, clean food and water. Initially, the feasible way to deal with these new scenarios and needs is to use expensive dedicated computing hardware with sufficient storage capacity. This stage lasted until the first decade of the 21st century, and the computing model has experienced three main models, namely, the mainframe centralized computing model, the personal computer distributed computing model, and the Client/Browser/Server model. With the expansion of urban scale and the rapid increase of various human demands, as well as the reduction of expensive investment in specialized hardware, the cloud computing model has been invented [5]. Cloud computing provides Information Technology (IT) resources, including servers (virtual servers and physical servers), data storage, networking, development tools, and analytic, on a pay-as-you-go mode via Internet. The striking features of cloud computing include flexibility, elasticity, reliability, and sustainability [6,7]. Although cloud computing provides economic and high-quality services for smart cities, its shortcomings, such as data security, high latency, non-context-aware, and poor seamless mobility support, are gradually exposed in the application of smart cities, especially in the context of Internet of Things (IoT) [8,9]. To solve these shortcomings of cloud computing, edge computing is proposed. As a new computing paradigm, edge computing integrates computing, storage and network resources placed at the edge of the network, in close proximity to the terminal devices and data, and provides real-time, low latency and high data security services [10]. Figure 1 illustrates the development roadmap of the main ICT computing models, as well as points out the main features, advantages and disadvantages of each computing model.

The relationship between edge computing and cloud computing is not substitution, but collaboration. Edge computing and cloud computing also have their own advantages. Cloud computing is good at global, non-real-time and long-term big data processing and analysis, and can give full play to its advantages in the fields of long-term maintenance, business decision support and other fields [11,12,13,14]. While edge computing is more suitable for the processing and analysis of local, real-time and short-term data, and can better support the real-time intelligent decision-making and execution of local business [15,16,17,18]. Table 1 compares the features, advantages and typical application scenarios of cloud computing and edge computing. As a new computing paradigm, the cloud-edge collaboration combines the respective advantages of the cloud computing and the edge computing, which can better improve the Quality of Service (QoS) of digital services, and has become a new research trend [19].

In the literature, several studies have been conducted to explore the cloud-edge collaboration, which can be divided into two categories. One is to study specific topics of cloud-edge collaboration, but lacks in-depth research on the content and challenges of cloud-edge collaboration reference model. For example, to address the problem of discovering and updating the physical topology of the large-scale IoT systems in real-time, a fast hierarchical topology update scheme by using the edge-cloud collaborative architecture is proposed [20]. Ref. [21] proposes an mobile edge-cloud computing resource allocation algorithm, which enables the edge and the cloud to share their computing resources in the form of wholesale and buyback. Ref. [22] proposes a novel collaborative clustering parallel Q-learning algorithm to realize high accuracy physical-cyber Digital Twin (DT) mapping in manufacturing systems. The other is the research on cloud-edge relationship and collaboration framework. For example, in [19], a cloud-edge collaborative Convolutional Neural Network (CNN) framework is proposed for cognitive service. Ref. [23] conducts a systematic review for the collaborative learning mechanism for cloud and edge modeling. Ref. [24] discusses the implementation principles and the process of cloud-edge collaboration technology.

The governance of smart cities is a highly complex and dynamic urban sociotechnical system, while smart governance with clouding computing and edge computing is a key element of smart cities [25,26]. In the literature, many applications of municipal service platform based on cloud computing and edge computing have been proposed [27,28,29]. Meanwhile, scholars begin to explore the applications of cloud-edge collaboration in smart manufacturing [22,30], smart transportation [31,32], smart health [33,34], content delivery [35], etc. However, the reference model and application instance based on cloud-edge collaboration specially for municipal service platform is rarely studied.

The optimal allocation of computing and communication resources in cloud-edge system has been attracted a lot of attentions [36,37], expecially in the Multi-access Edge Computing (MEC) scenario [38,39]. In general, the optimization goal is to minimize the energy consumption, offloading delay, and the total cost of the cloud-edge system. However, in the cloud-edge system composed of Multiple Service Providers (MSPs) and Multiple Service Demanders (MSDs), the current optimization schemes rarely considered the dynamic competition among the MSPs. In addition, the evolution of MSDs to achieve the maximum utility by offloading their tasks to different MSPs was also ignored [40].

The main contributions of this paper are as follows.

(1) We develop an reference model based on cloud-edge collaboration specially for municipal service platform. We point out the main content and challenges of cloud-edge collaboration, including resource collaboration, application collaboration, service collaboration, and security collaboration.
(2) We design and implement a smart e-government self-service system based on the above reference model. The experimental results show that the cloud-edge collaboration mode can provide a better user experience.
(3) We propose a transaction model to study the optimal allocation strategy for the optimal allocation of computing resources and communication resources in the cloud-edge collaboration. The findings show that the evolutionary and the Nash equilibrium are the optimal solutions, respectively.

The rest of this paper is organized as follows. Section 2 discusses the content and the challenges of the cloud-edge collaboration. Section 3 introduces a game-theory-based dynamic resource allocation model. Section 4 develops an reference model, and then designs and implements a smart e-government self-service system based on this model. Finally, Section 5 summarizes the whole paper.

## 2. Cloud-Edge Collaboration

Effective cloud-edge collaboration requires deep integration of disparate infrastructures and systems to seamlessly migrate and execute services according to business needs, which is a very challenging work. In general, from the perspective of collaboration mode, cloud-edge collaboration can be divided into two categories: cloud-to-edge (C2E) collaboration and edge-to-edge (E2E) collaboration. In C2E mode, the cloud is mainly responsible for tasks such as big data analysis, model training, and strategy update and distribution, while the edge is responsible for local data collection, transmission, strategy implementation, and feedback. In E2E mode, the edge devices located in different locations realize data sharing and collaborative work by establishing a secure communication mechanism. This collaborative mechanism improves the quality and speed of application services, as well as realizing regional autonomy while protecting data privacy.

In 2020, a study released by the Edge Computing Alliance and Industrial Internet Alliance (ECAIIA) points out that the cloud-edge collaboration can be divided into three categories from the perspective of the IT infrastructure framework: resource collaboration, application collaboration, and service collaboration [41]. However, as an important issue, security collaboration is ignored. As a new computing paradigm, cloud-edge collaboration shres with security threats of cloud computing and edge computing, while includes its new peculiar ones [42]. This section discusses the main content and challenges of these four collaboration categories.

### 2.1. Resource Collaboration

Resource collaboration refers to the unified management of hardware and software resources of edge nodes and cloud servers by deploying resource scheduling management interfaces in edge nodes and cloud servers, respectively. Resource collaboration schedules cloud computing and edge computing resources from a global perspective, which greatly improves the efficiency of resource utilization. However, resource collaboration also faces the following challenges.

(1) Heterogeneous and diverse edge devices. Take autonomous driving as an example, the autonomous driving system covers three scenarios: (a) the system senses the surrounding environment of the vehicle through various sensors, such as laser radar, millimeter wave radar, ultrasonic radar and camera. (b) based on the perceived information, the processor makes corresponding decisions and sends control signals to the actuator. (c) The actuator receives decision signals and takes corresponding actions, such as braking, warning, etc. In the above mentioned process, there are various types of hardware and computing architectures involved. For exmaple, the mainstream processor manufacturers of autopilot system include MobilEye, Nvidia, Qualcomm, Infineon, Xilinx, etc. The processors include MobilEyeQ5, Nvidia drive Orin and Zynq®-7000 All Programmable SoC and so on, while the computing unit architecture includes Central Processing Unit (CPU), Graphics Processing Unit (GPU), Digital Signal Processor (DSP), Field Programmable Gate Array (FPGA), Application Specific Integrated Circuit (ASIC), etc. At the same time, the rapid development of technology has led to the coexistence of multiple manufacturers and multiple generations of technologies in one product life cycle. In general, there are huge differences in computing power, communication resources, technical solutions among these heterogeneous edge devices. In practical applications, the same code cannot be directly deployed to different devices. Therefore, the compatibility interface design and the reasonable management and allocation of resources among heterogeneous devices become a very challenging task.

(2) Resource-constrained edge devices. With the development of high and new technologies such as AI, IoT, and new materials, smart terminals have evolved rapidly. Their product forms have become more and more diversified with increased capabilities and intelligence, from hand-held smartphones to wearable smart devices and sensors. In smart traffic management, various ambient sensors, such as cameras and radars, are installed on streets and crossroads to monitor traffic conditions in real-time, and these collected historical data are used to optimize traffic control. Especially, when necessary, cameras equipped with AI chips are used to track the public and identify specific people by recognizing facial or physical features automatically and efficiently, thereby improving public safety. The ever-growing smart cities applications, such as smart meters, transportation, package or asset tracking, and video surveillance, are contributing in a major way to the growth of smart terminals and connections. According to Cisco Annual Internet Report (2018–2023) white paper [43], there will be 14.7 billion Machine-To-Machine (M2M) connections by 2023, up from 6.1 billion in 2018, with an amazing Compound Annual Growth Rate (CAGR) of 15.8% over the forecast period (2018–2023). In particular, the global mobile devices will reach 8.8 billion by 2023, of which 1.4 billion will have 5G capability. Edge devices are embedded devices or small devices with limited computing, power, memory and network connectivity. In order to achieve business goals, computing offloading needs to be considered, that is, computing tasks are carried by the edge or the cloud [44]. If edge applications need to implement elastic architecture or failover similar to cloud computing, a cluster of multiple edge nodes is required to deploy, schedule, and manage applications in this cluster.

(3) Complex network communication. Cloud-edge collaboration needs to span multiple types of communication networks, such as cellular networks, Wi-Fi networks, and Low-Power Wide-Area (LPWA) networks. Cellular networks are evolving from lower-generation network connectivity (i.e., 2G) to higher-generation network connectivity (i.e., 3G, 4G and now 5G), and are mainly used for indoor and outdoor voice communication and outdoor multimedia applications. Statistics from a report of Cisco shows that, currently, 4G is the predominant mobile network and the global 4G connections will grow from 3.7 billion in 2018 to 6.0 billion by 2023 with a CAGR of 10 percent [43]. Compared with cellular networks, Wi-Fi networks restrict the main usage scenarios to indoor by limiting the transmit power of the Access Point (AP), and are used for bandwidth-hungry and latency-sensitive applications, such as virtual reality, high-definition video, and video surveillance. Currently, Wi-Fi networks are widely used in the form of hot-spots in the dense environment of smart cities, where there are many concurrently connecting devices and IoT connections, such as airports, public transportation, retail, healthcare, stadiums, etc., supporting large-scale IoT connection density, as well as highly interactive and tactile applications. Globally, there will be nearly 628 million public Wi-Fi hot-spots by 2023, up from 169 million hotspots in 2018 with a fourfold increase [43]. LPWA is specifically designed for M2M usage scenarios. From the perspective of coverage distance, the mainstream LPWA technologies can be divided into two categories. One category includes NB-IoT [45], Sigfox [46], and LoRa [47], which are mainly used in long-distance communication scenarios. Examples include smart sensors for automatic inventory checking, gas or water meters in residential basements, and pet or personal asset trackers. The other includes Near Field Communication (NFC) [48], Radio Frequency Identification (RFID) [49], Bluetooth [50], and Zigbee [51], which are mainly used in short-distance communication scenarios, such as mobile payment, home automation, and personal consumer electronics. Different networks have different bandwidth, propagation delay, and link quality. Therefore, how to realize the communication link with low delay, high quality and low cost is an extremely challenging technical task.

### 2.2. Application Collaboration

Application collaboration means that users can remotely deploy edge applications to any desired edge nodes through the cloud server to provide services for terminal devices, and manage the life cycle of edge applications through the cloud server. Application collaboration can greatly improve the deployment efficiency of edge applications and reduce operation and maintenance management costs. However, in specific practice, application collaboration faces the following challenges.

(1) The differences in edge computing scenarios and hardware infrastructures bring the application deployment challenge. As in the previous example of autonomous driving, the types of hardware and computing architectures are diverse. At the same time, the rapid development of technology has led to the coexistence of multiple manufacturers and multiple generations of technologies in one product life cycle.

(2) Large-scale distribution of applications to massive edge devices brings application distribution challenge. For example, assume that there are 100,000 edge nodes need to download application images for deployment. Each application image file is 100 MB, then the total data volume is 10,000 GB, which brings high performance and bandwidth requirements to the cloud central image server. In addition, in many application scenarios of edge computing, such as smart grid, smart traffic management, and smart street lighting, the edge nodes and the cloud central mirror server generally span a variety of network connections, such as the Internet, mobile communication network, NB-IoT, etc., resulting in relatively poor network stability. Frequent network disconnection not only greatly affects the deployment of applications on edge nodes, but also affects the operation and maintenance of applications. Therefore, in the offline scenario, the autonomous management of edge node applications is also a great challenge. In short, these have brought great challenges to the business choreography, hardware framework and application deployment, and application life cycle management of application collaboration.

### 2.3. Service Collaboration

Service collaboration refers to the mechanism of docking and adapting edge services of edge computing and cloud services of cloud computing to quickly build edge applications and provide services for edge users. Service collaboration can help user quickly access, discover, operate, and maintain edge services by utilizing the existing cloud service architecture. Many cloud service providers have proposed collaborative services specifically for edge computing. For example, Google has deployed more than 1400 edge servers worldwide and proposed solutions for edge applications. Amzaon CloudFront has provided low-latency content delivery network services required by edge computing applications. Alibaba has deployed more than 500 edge servers in China, enabling users to obtain low-latency edge service anytime, anywhere via collaborative services [52]. However, service collaboration also faces the following severe challenges.

(1) The resources of edge devices are limited, while the amount and types of data on the edge side are increasing at an alarming rate, which poses challenges to the real-time and reliability of edge computing. For example, unlike traditional data, real-time monitoring sensors generate a large amount of time-series data all the time. These data are time-tagged, chronologically changing, including device status, metadata, sensor status, etc.

(2) The diversification of edge services leads to the diversification of development frameworks and algorithms, which increases the difficulty of service collaboration. For example, in the field of video surveillance, autonomous driving, and VR, many data to be processed are unstructured data. These types of data have the characteristics of huge volume data, high real-time requirements, and large computing resource consumption. The AI development framework includes tensorflow, pytorch, mindspore, etc. AI technologies include deep learning, incremental learning, federated learning, efficiency-primary collaboration, transfer learning, meta-learning, edge graph neural networks, and edge-cloud reinforcement learning [23]. However, in edge computing scenarios such as industrial control and automated production, the data are dominated by structured data, and traditional machine learning algorithms are mainly used, such as decision tree, support vector machine, logistic regression, etc. All these have brought great challenges to the data, algorithms, communication protocols and interface library of service collaboration.

### 2.4. Security Collaboration

Cloud-edge collaboration extends data from the cloud to the user end and data production end, as well as provides the possibility for various applications to share computing results. On the contrary, These conveniences pose a great threat to the security and privacy [42]. In general, as an extension of cloud computing and edge computing, cloud-edge collaboration faces the same security issues and threats as both of them.

In the literature, many studies have focused on the security of cloud computing and edge computing [53]. According to a case study released by the Cloud Security Alliance (CSA), the top three threats in cloud computing are data breach, insufficient identity, credential and access management, and insecure interfaces and Application Program Interfaces (APIs) [54]. Cloud virtualization security [55,56], cloud data security [57,58,59], and cloud application security [60,61] are also widely discussed. Other recognized issues related to security and privacy in cloud computing include multi-tenancy, confidentiality, and phishing [62]. Meanwhile, the security issues discussed in edge computing are mainly divided into the following four topics: access control [63,64], identity authentication [65,66], data security [67,68], and privacy [69,70].

As a new computing paradigm, cloud-edge collaboration also faces its new security issues, but rarely studied in previous literature. According to our research and development practice, we believe that cloud-edge collaboration faces the following security challenges.

(1) Challenge of APIs security. Edge devices and cloud servers need to work together through a large number of APIs, which increase the risk of illegal access. For example, developers may expose all their object properties without considering the security sensitivity of these properties, which easily leads to excessive data exposure challenge. Imperfect API authentication is another example of potential challenge. If the authentication implementation scheme of the cloud-edge system is not perfect, attackers can impersonate an API legitimate user to access confidential data.

(2) Challenge of designing security features on resource-limited edge devices. Developing universal security functions for a large number of heterogeneous edge devices is a great challenge. On the other hand, adding design security features to resource-constrained edge devices increases the complexity of software and the risk of system failure.

(3) Device authentication is an effective measure to verify the valid identity of the access device and reduce the risk of being attacked. However, in edge computing scenarios, many edge devices do not have enough storage and computing resources to perform cryptographic operations required by authentication protocols. In addition, it is a difficult task to realize unified identity authentication and key management of massive edge devices in large-scale, heterogeneous and dynamic edge networks. Furthermore, it is also a great challenge to provide access control functions for large-scale dynamic and heterogeneous edge devices, and to support the distributed remote periodic update of user information and security policy information.

## 3. Dynamic Resource Allocation Model

In the cloud-edge collaboration scenario, the computing and communication resources of cloud server are limited. How to optimally allocate resources is a challenging task, especially when considering the competition between edge devices. In our previous research, we propose a transaction model to study the optimal allocation strategy of computing and communication resources in MSPs and MSDs system [40]. The MSPs and MSDs model is the most universal model. The cloud-edge collaboration system of one cloud server and multiple edge devices designed in Section 4 can be regarded as a special case of this model. The following is a brief introduction to this transaction model.

### 3.1. MSPs Model

The satisfaction of MSPs is related to the number of resources they have, which can be quantified as the following logarithm function.

(1) $$O\left(C,B\right)=u_{2}log\left(k_{1}C+k_{2}B\right)$$

where *C* is the number of CPU cycle and $k_{1}$ is the computing efficiency in bits per symbol. $k_{2}B$ is the bandwidth. *B* is the size of spectrum and $k_{2}$ is the spectrum efficiency in bits per symbol due to adaptive modulation. $u_{2}$ is a constant depending on the application type.

In a real-world scenario, the SPs can adaptively adjust the number of resources sold to SDs according to the competition with other SPs and the needs of SDs. Its benefit function can be defined as:

(2) $$Z_{j}\left(b_{j},c_{j},v_{j}\right)=O\left(C_{j}-c_{j},B_{j}-b_{j}\right)+\sum l\in \Lambda g_{l,j}v_{j}$$

where $B_{j}$ and $C_{j}$ are the total number of CPU cycle and spectrum size owned by SP *j*. $b_{j}$ and $c_{j}$ are the portion of CPU cycle and spectrum offered to SDs by SP *j*. $v_{j}$ is the price set by SP *j* for resources $b_{j}$ and $c_{j}$, and Λ is the index set of groups whose SDs purchase resources from SP *j*.

### 3.2. MSDs Model

The satisfaction function $S_{i,j}$ for SD *i* to purchase resources from SP *j* can be defined as follows.

(3) $$S_{i,j}=u_{1}log\left(\frac{c_{i,j}}{f_{j}}-\frac{1+\beta_{i}}{b_{i,j}log_{2}\left(1+\frac{p_{i}h_{i}}{N_{0}}\right)}\right)$$

where $c_{i,j}$ is the number of CPU cycle purchased from SP *j* by SD *i*, and $f_{j}$ is the number of CPU cycle required to compute 1-bit data for SP *j*, which represents the computing speed of SP *j*. $p_{i}$ is the transmission power for SD *i*, $N_{0}$ is the noise power spectrum density, and $h_{i}$ is the channel gain between the SP and SD *i*.

### 3.3. Formulation of the Evolutionary Game

Multi-population dynamic evolutionary game theory is used to model the selection behaviors of SDs. The optimal solution of the evolutionary game is called evolutionary equilibrium. At the evolutionary equilibrium point, SDs stop making changes. The benefit function of SD in group l purchasing resources from SP *j* can be defined as follows.

(4) $$V_{l,j}=u_{1}log\left(\frac{c_{j}}{f_{j}\sum l\in \Lambda g_{l,j}}-\frac{\left(1+\beta_{l}\right)\sum l\in \Lambda g_{l,j}}{b_{j}log_{2}\left(1+\frac{p_{l}h_{l}}{N_{0}}\right)}\right)$$

Based on the above three models, we model the dynamic behaviors of MSDs using an evolutionary game, and then we design the stochastic and deterministic iterative algorithms to study the evolution of MSDs. The findings show that evolutionary equilibrium is the optimal solution. Furthermore, we adopt the non-cooperative game method to model the competition among MSPs, and propose an iterative algorithm to obtain the optimal solution, which is the Nash equilibrium.

## 4. Design and Implementation of E-Government Self-Service System

Williamson suggests that governance is the means to achieve order in a relationship in which potential conflicts may undermine or upset opportunities for the realization of mutual gains [71]. In the field of urban governance, modern city is a highly complex and dynamic urban sociotechnical system. The conventional governance and management approaches have encountered great challenges. However, technological innovations, especially in the ICTs, can govern the inherent complexity of large urban system in a more effective way. As a result of our theoretical research and practice, we point out that people-oriented is one of the basic principles of smart city governance [4]. In this paper, we argue that the so-called smart governance refers to the use of edge computing, big data, cloud computing, AI and other cutting-edge technologies to enable citizens not only express their voice freely to the stakeholders, but also participate in the management of urban affairs more effectively. Putting the interests of urban residents in the first place is a common purpose of using these sophisticated technologies. In this research work, we cooperated with B.S. Information Technology Co., Ltd. to develop an e-government self-service system based on the cloud-edge collaboration. This section describes the user requirement analysis, reference model of cloud-edge collaboration, and edge self-service terminal design. Specifically, the design of edge self-service terminal mainly refers to and follows the design specifications for edge devices issued by Open Data Center Committee (ODCC) [72].

### 4.1. User Requirement Analysis

Urban residents need to deal with various municipal businesses, which can be classified into identity authentication and identification, business inquires, Business declaration and payment. Specifically, these businesses include medical insurance reimbursement, housing transaction, Payment of traffic fines, payment of resident endowment insurance, etc. These businesses are closely related to residents’ lives, but distributed in different urban management departments. It is inefficient for residents to carry paper-based certificates and business materials between departments when dealing with their businesses. When the business handled involves multiple departments, the entire process becomes more tedious. The requirements of urban residents can be summarized as follows.

(1) The system can comprehensively handle the business of urban management departments and break the data isolated islands among these departments.
(2) Urban residents can easily access the system and realize municipal business “one-stop” service through self-service. These municipal businesses include identity authentication and identification, business query, Business declaration and payment.
(3) The municipal business services provided should be flexible, easy to expand and uninstall according to business needs, as well as meet relevant security requirements.

### 4.2. System Architecture Design

**Figure 2:**
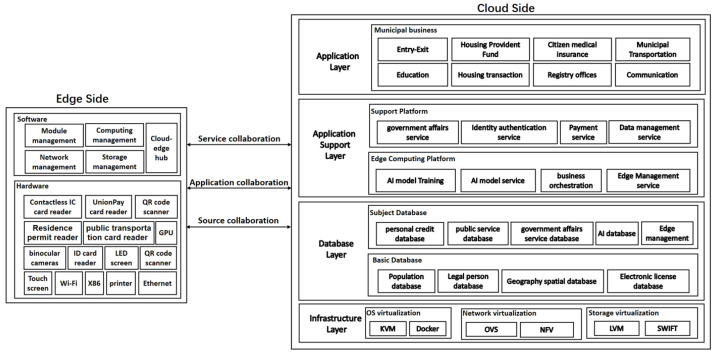
The cloud-edge collaboration reference model.

The system adopts C2E collaboration architecture design. The cloud side is the server, and the edge side is the self-service terminals with AI embedded. By collaborating on resources, applications and services, the system facilitates citizens’ one-stop self-service e-government. The cloud-edge collaboration reference model of e-government self-service system is shown in Figure 2.

In terms of resource collaboration, the self-service terminal provides computing, storage, connection, networks, virtualization and other infrastructure resources required for service, such as user authentication, user information reading and writing, while the cloud server provides self-service terminal device management.

In terms of application collaboration, the cloud server uniformly deals with the business process orchestration logic of municipal services, trains high-precision AI recognition models, and quickly updates the business and AI models to a large number of self-service terminals through the application distribution interfaces between cloud and edge.

In terms of service collaboration, the edge device decides whether to process the business locally or upload to the server according to the collected user information and business processing requirements. The edge device receives the processing results of the server, updates the local data, and prints the business processing results. When the edge AI model finds an unrecognized target, it collects images and uploads them to the cloud. The cloud makes further judgment through the high-precision model, and sends the results to the edge devices. The edge side regularly uploads the collected pictures, videos and other feature information to the cloud for model training.

### 4.3. Edge Self-Service Terminal Design

**Figure 3:**
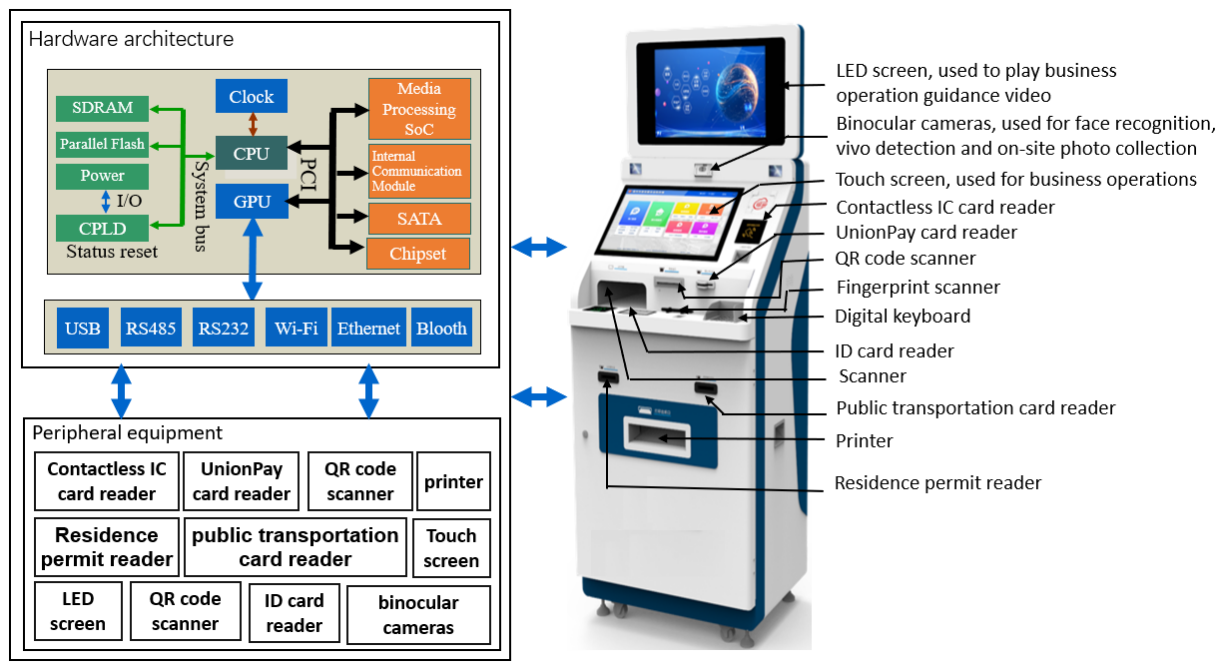
The hardware framework of self-service terminal.

The main board of the self-service terminal adopts industrial control computer, and the working temperature ranges from 0° to 60°. The key features include a high-performance 64-bit multi-core processor Intel^®^ i7-10700, Intel chip-set H420E, dual-display support at resolutions up to 4 K via a pair of micro-HDMI ports, hardware video decode at up to 4Kp60, up to 32 GB of dual channel DDR4 ARM, up to 1T SSD hard disk, dual-band 2.4/5.0 GHz wireless LAN, Bluetooth 5.0, Gigabit Ethernet, USB 3.0, PCI-E, RS232, and PoE capability. Through the above different interfaces, the motherboard is connected with input and output devices such as led display, binocular cameras, touch screen, contactless integrated circuit (IC) card reader, union-pay card reader, quick response (QR) code scanner, fingerprint scanner, identity card reader, public transportation card reader, printer, residence permit reader, and so on. The terminal runs Windows 10 operating system. Figure 3 shows the hardware framework of self-service terminal.

### 4.4. Result Analysis

**Figure 4:**
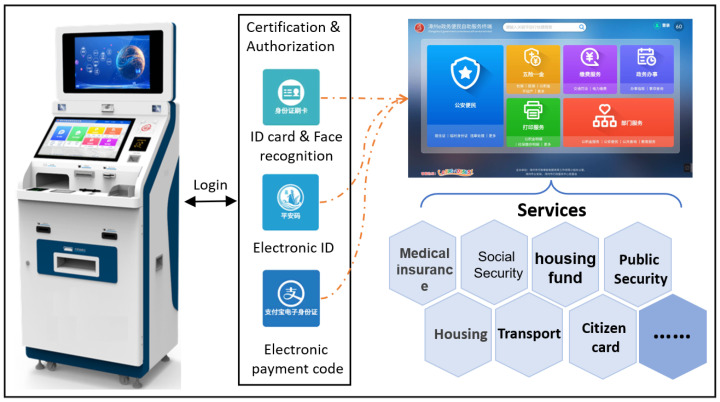
The running interfaces of system (This system is developed for the Zhangzhou Municipal Government).

The cloud server is deployed in the data center of e-government extranet, which meets the three-level security protection requirements of the Information Security Classified Protection Standard (ISCPS) issued by ministry of public security of China. The self-service terminals are deployed in the municipal hall of the citizen service center, and connected to the e-government extranet through the Internet to communicate with the cloud server. The running interfaces of system are shown in Figure 4.

In this paper, we selected three major services in e-government, namely, face recognition, video decoding and service processing, and then compared and analyzed the differences between edge computing and cloud computing from the aspects of latency, bandwidth, single task performance, and multi-task concurrency performance.

In the comparative test of latency and bandwidth, the test method is to test each service 10 times and then calculate the average value. The experimental results show that edge computing has obvious advantages, with the latency reduced by two-thirds and the bandwidth increased by five times, as shown in Table 2. In the first test, the business needs to be processed in the cloud, there is no obvious difference in the latency of the two computing models. However, in the subsequent tests, since the edge computing caches the above business locally for processing, the latency shows an obvious difference. Since edge computing concentrates services on the local area network, while cloud computing needs to go through the Internet and e-government extranet, the bandwidth is obviously limited.

**Figure 5:**
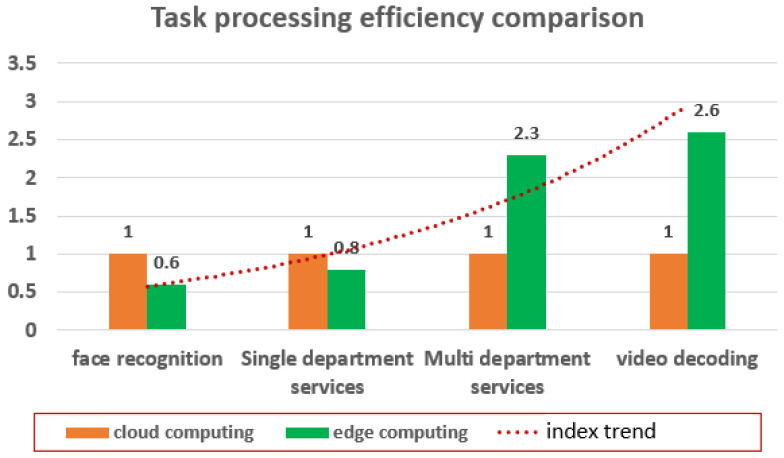
Task processing efficiency comparison.

In the comparative test of single task performance, the test method is to run a service completely, record the service completion time, repeat the test for 10 times, and calculate the average service completion time. The results are normalized and compared based on the task processing time of cloud services. The experimental results show that the two computing models demonstrate obvious differences for different services, as shown in Figure 5. For multi-department services and video decoding, cloud computing has distinct advantages over edge computing. The reason is that video decoding requires a lot of computing and storage resources, and cloud computing offers more benefits. As for multi-department services, since businesses are all deployed on the cloud platform, multiple business interactions can be completed internally at one time, while edge computing needs multiple interactions with the cloud server. For localized services, such as face recognition and single department service, edge computing has higher bandwidth and lower latency.

**Figure 6:**
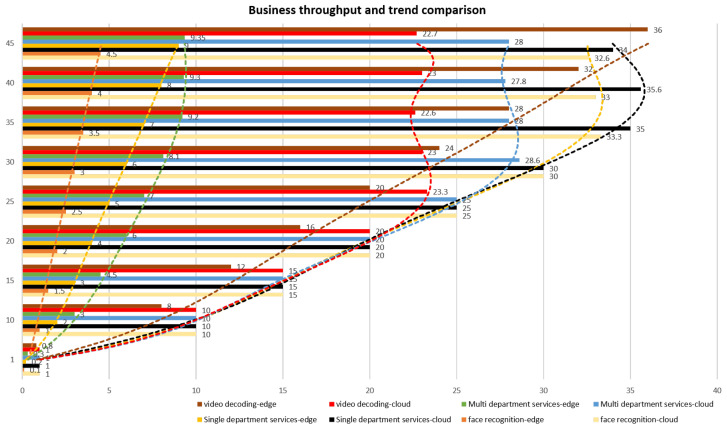
The business throughput and trend comparison.

In the system throughput comparison test, the test method is to run a service separately, gradually increase the number of users in a multiple of five, and record the maximum number of services completed by the system in one second. In practice, the working mode of self-service terminal is single-user & multi-task. Therefore, we scale the number of terminals and the number of concurrent test users in the 1:1 relationship. Furthermore, in order to better distinguish the test results, for the throughput test of each service, we use the polynomial fitting method to draw the trend curve of throughput. The experimental results show that the business throughput trend curve of edge computing paradigm is close to linear growth. While The trend curve of cloud computing paradigm starts with the rapid growth of throughput with the increase of the number of concurrent users. After reaching the peak, it decreases and fluctuates continuously with the increase of the number of concurrent users, as shown in Figure 6. In practice, the self-service terminal is a single user system. For services processed by the self-service terminal, such as face recognition and single department service, the throughput increases approximately linearly with the increase of users. However, as the previous theoretical analysis shows, for businesses involving edge computing and cloud computing collaboration, such as multi-department services, there will be peaks and fluctuations. It should be noted that in practice, the self-service terminal is a single-user system. For services processed at the self-service terminal, such as face recognition and single department service, the throughput increases approximately linearly with the increase of users. However, as shown in the previous theoretical analysis, businesses involving edge computing and cloud computing collaboration, such as multi-department services, will have peaks and fluctuations.

## 5. Conclusions

In this paper, we propose a cloud-edge collaboration reference model and application instance of this model, an e-government self-service system. The reference model comprehensively discusses the content and challenges of cloud-edge collaboration from resource collaboration, application collaboration, service collaboration, and security collaboration. The experimental results indicate that the designed system provides better user experience. In addition, this paper presents a game-theory-based dynamic resource allocation model for computing and communication resources allocation in the cloud-edge collaboration. The research results show that the evolutionary equilibrium and the Nash equilibrium are the optimal solutions, respectively. We hope that this study can provide useful reference for decision makers and builders of smart cities. Future works may further consider the real-time performance and computational complexity of the proposed system at a practical level. In addition, as one of the key issues, the security of cloud-edge collaboration also needs further consideration.

## Figures and Tables

**Table 1:** Comparision of the cloud computing and the edge computing.

Features	Edge Computing	Cloud Computing
computing paradigm	Provides distributed nearby computing and storage services at the edge of the network, focuses on the analysis of real-time and short-term data, and supports the real-time intelligent processing and execution of local business better.	Provides centralized on-demand remote computing and storage self-services in the network center, focuses on the analysis of non-real-time and long-term big data, and provides better support for business decision-making.
devices	Embedded smart devices or credit-card-size computers with weak computing power, generally have waterproof and dustproof design. Not only data consumers but also data producers.	High performance server located in the data center with powerful and dynamically adjustable computing power.
advantages	Low latency, low energy consumption, high business security, and high personal privacy.	Self-service, resource pooling and sharing, elastic scaling, and service measurable.
typical scenarios	In the field of intelligent manufacturing, factories use IoT gateway, industrial robots, and sensors for data localization processing, such as data acquisition, filtering, cleaning and real-time control. Moreover, the IoT gateway provides the ability of heterogeneous protocol fusion and realizes the unified access of industrial networks. In the field of smart cities, managers realize the collection, analysis and real-time control of various data through various sensors, drones, cameras, and embedded computers. In the field of live games, edge computing provides Content Delivery Network (CDN) with rich storage resources and audio and video rendering capabilities closer to users, especially in Augmented Reality (AR) and Virtual Reality (VR) scenes.	Comprehensive big data applications. For example, the Hangzhou’s City Brain is a smart city cloud platform, which aims to improve the urban management by using of big data, cloud computing, AI, and other technologies. High-performance computing applications. For example, aerodynamic design and analysis, weather forecast and meteorological research, virtual simulation of digital twin cities, etc. Huge business volume fluctuation; applications with sudden increase of system load due to the unpredictable access volume of the client. Once the system load is too large, there may be problems such as system downtime, inaccessible services, and poor customer experience. Such cloud platforms include Amazon, Microsoft Azure, IBM Cloud, etc.

**Table 2:** Comparison of the latency and bandwidth.

Service Type	Latency/ms	Bandwidth/Mbps
Cloud computing	>=15	=<20
Edge computing	=<5	>=100

## Data Availability

Not applicable.
